# Prophage Induction Is Enhanced and Required for Renal Disease and Lethality in an EHEC Mouse Model

**DOI:** 10.1371/journal.ppat.1003236

**Published:** 2013-03-28

**Authors:** Jessica S. Tyler, Karen Beeri, Jared L. Reynolds, Christopher J. Alteri, Katherine G. Skinner, Jonathan H. Friedman, Kathryn A. Eaton, David I. Friedman

**Affiliations:** 1 Department of Microbiology and Immunology, University of Michigan, Ann Arbor, Michigan, United States of America; 2 Department of Mathwork, Mathworks, Natick, Massachusetts, United States of America; Tufts University School of Medicine, United States of America

## Abstract

Enterohemorrhagic *Escherichia coli* (EHEC), particularly serotype O157:H7, causes hemorrhagic colitis, hemolytic uremic syndrome, and even death. In vitro studies showed that Shiga toxin 2 (Stx2), the primary virulence factor expressed by EDL933 (an O157:H7 strain), is encoded by the 933W prophage. And the bacterial subpopulation in which the 933W prophage is induced is the producer of Stx2. Using the germ-free mouse, we show the essential role 933W induction plays in the virulence of EDL933 infection. An EDL933 derivative with a single mutation in its 933W prophage, resulting specifically in that phage being uninducible, colonizes the intestines, but fails to cause any of the pathological changes seen with the parent strain. Hence, induction of the 933W prophage is the primary event leading to disease from EDL933 infection. We constructed a derivative of EDL933, SIVET, with a biosensor that specifically measures induction of the 933W prophage. Using this biosensor to measure 933W induction in germ-free mice, we found an increase three logs greater than was expected from in vitro results. Since the induced population produces and releases Stx2, this result indicates that an activity in the intestine increases Stx2 production.

## Author Summary

Infection with Enterohemorrhagic *E. coli* (EHEC), and more recently with the Enteroaggregative *E. coli* strain O104:H4, is a significant health risk, causing bloody diarrhea, kidney failure, and even death. The virulence factor in these bacteria responsible for the severe outcomes is Shiga toxin (Stx). Genes encoding Stx are in the genome of bacterial viruses (prophages) on the pathogenic *E. coli* chromosomes. The prophage remains quiescent until damage to the bacterial chromosome occurs causing prophage gene expression (called induction), which leads to production of bacteriophages that are released into the environment. Because *stx* expression is controlled by the phage regulatory system, prophage induction leads additionally to production and release of Stx. This study provides conclusive evidence that in a mouse model of EHEC infection, induction of the prophage carrying the *stx* genes is specifically required for EHEC to cause disease and that the intestinal environment adds to the induction and therefore to the production of Stx. Similar events likely regulate Stx production and release by the Stx encoding phage in the O104:H4 strain. Controlling prophage induction offers a means to control EHEC infection.

## Introduction

Enterohemorrhagic *E. coli* (EHEC) has emerged as a serious health threat with numerous outbreaks most commonly due to contaminated beef, but also to contaminated vegetables and water [1]. Although EHEC strains [2], and another recently identified pathogenic *E. coli* [3], encode a number of virulence factors, the most serious sequelae of infection by these strains are due to the acquisition and expression of genes encoding Shiga toxins (Stx).

In many EHEC strains these toxins are encoded in the genomes of prophages of the λ family (referred to as lambdoid phage) [4]. Two major classes of Shiga toxins, Stx1 and Stx2, have been identified in EHEC strains [5]. Although sharing the same activity, they differ somewhat in sequence and Stx2 is associated with the more severe sequelae in humans [6] and is the cause of disease in animal models [7]. These members of the AB_5_ class of toxins bind eukaryotic cells by attachment of the pentameric structure of the B subunit to a glycoprotein receptor on the eukaryotic cell [8]. Retrograde transit through the endosomic pathway to the cytosol results in the A subunit, a glycosidase, reaching the ribosomal RNA [9]. There, a specific adenine residue in the large ribosomal subunit is cleaved, resulting in arrested protein synthesis that leads to cellular intoxication [10]. EHEC strains commonly isolated in outbreaks are those of the O157:H7 serotype [6].

Members of the lambdoid family of temperate phages share a common genome organization with prototypical λ. Genes at the same relative position on their respective genomes may differ in sequence, but for the most part they share the same activity [11]. For example, the repressors and operators may differ in sequence and specificity, but the different lambdoid phages have a common structure and location for these genetic elements on their genomes [12]. Moreover, the lambdoid phages are mosaics with each phage sharing a number of different genes with different members of the family [11], [13]. These conserved structure-function relationships allowed for the relatively rapid determination of the role of the phage in Stx expression [14].

**Figure 1:**
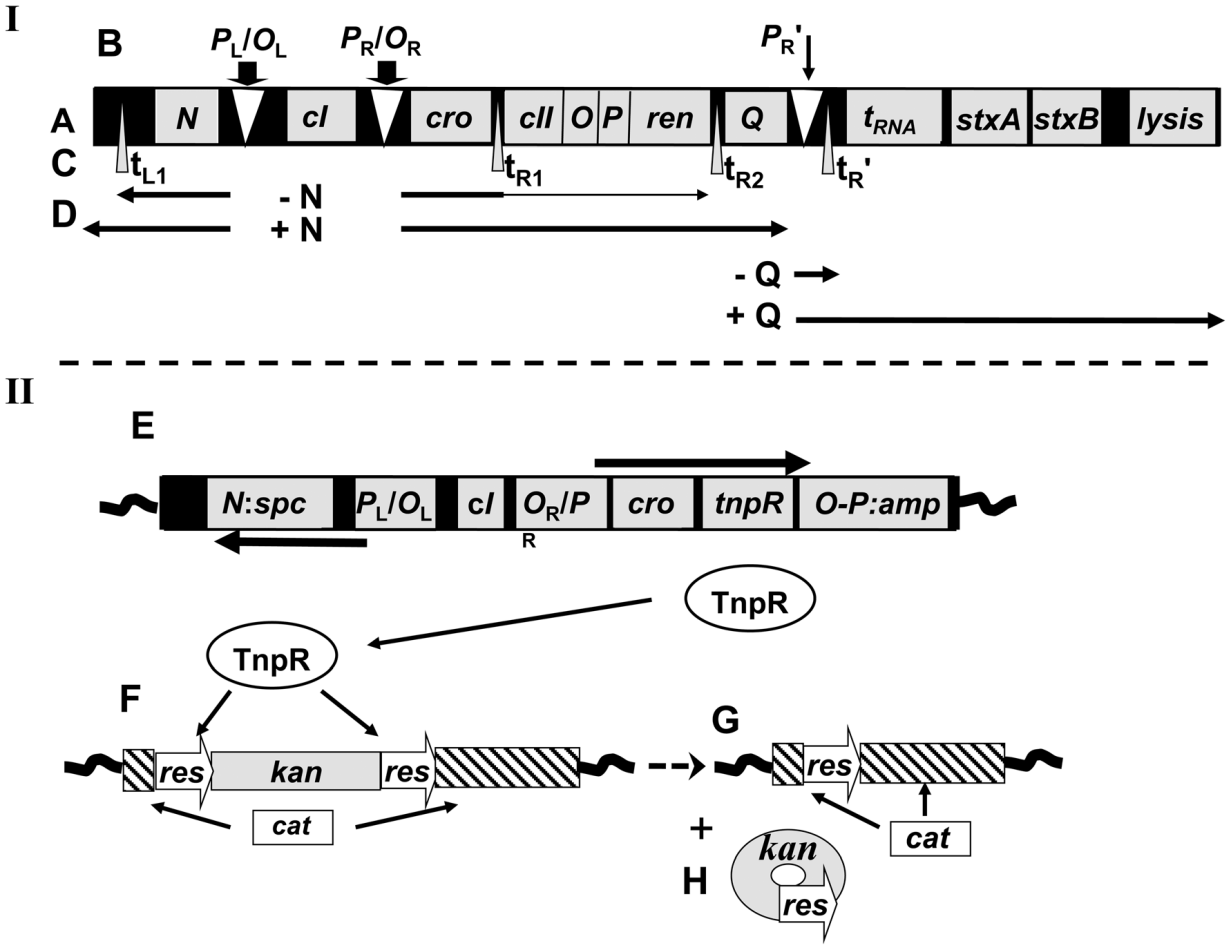
Diagrams illustrating genetic arrangements and constructs. **I.** The regulatory region of *stx*-carrying lambdoid phage and patterns of regulated transcription (not drawn to scale) (A) Arrangement of relevant genes. (B) Operators and promoters. (C) Transcription terminators. (D) Transcription patterns based on studies with λ. Temporal order: Early transcripts from *P*_L_ and *P*_R_ ending at indicator terminators, with ∼40% transcending t_R1_. Action of antitermination protein N allows synthesis of delayed early transcripts. Action of antitermination protein Q immediately downstream of *P*_R_′ allows maximum synthesis of late transcripts that include the *stx* genes. **II.** Components of SIVET (not drawn to scale). Top: (E) The early regulatory region of the 933W SIVET prophage. Deletion substitutions, *N*::*spc* and *O-P*::*amp* eliminate lethality by the induced prophage. The *tnpR* gene encodes the γδ resolvase and is transcribed from the prophage *P*_R_ promoter and under control of the 933W repressor. Hence, TnpR is not expressed by the repressed lysogen, but is expressed when induction leads to autocleavage of the phage repressor. Bottom: (F) SIVET reporter cassette with the *cat* gene interrupted with a *kanR* cassette flanked by *resC* sites (targets for TnpR resolvase). In this form the reporter cassette confers KanR. (G) Action of TnpR removes the *kanR* cassette and one flanking *resC* site. This leaves the *cat* gene interrupted by one *resC* site on the bacterial chromosome. By proper designing the position of the insertion site of the cassette in the *cat* gene of the reporter cassette as well as engineered small nucleotide changes in the *res* sequence, excision of the *kanR* cassette leaves a functional *cat* gene even though a *resC* sequence remains within the *cat* gene (confers CamR) [47]. (H) The *kanR* cassette with the other *resC* site is excised as a non-replicating circle and lost by segregation. The thick horizontal arrows in E represent patterns of transcription from early promoters following induction, loss of repression.

When present, the *stxA* and *B* genes are located downstream of *P*_R_′, the late phage promoter [15], [16], and upstream of the phage lysis genes (Fig. 1) [14], [17]. In vitro and in vivo studies with the O157:H7 strain 1∶361 and its resident *stx2*-phage, φ361, showed that transcription from *P*_R_′ is required for Stx2 production [18]. In vitro studies with the *E. coli* strain K9675 (a derivative of the nonpathogenic strain K37 lysogenized with the *stx2*-phage 933W) showed that Stx2 expression requires prophage induction [19]. Hence, Stx2 expression, at least under these in vitro conditions, depends on the phage induction cascade. Prophage induction explains why patient treatment with antibiotics that can act as inducing agents, such as the quinolones, lead to higher Stx levels [20] and exacerbate the disease [21].

The lambdoid phage regulatory cascade which leads to phage production and cell lysis has been the subject of years of study with λ and to a lesser extent with other members of this family of phages [22]. Induction, which results in the initiation of the regulatory cascade, is set in motion when the bacterium containing the prophage (lysogen) sustains DNA damage and responds by activation of the LexA regulon, leading to a cellular change in gene expression termed the SOS response [23], [24]. One member of this regulon, RecA, increases in quantity and assumes an activated form, RecA*, by interacting with single-stranded DNA generated by DNA damage [25]. RecA*, through its co-protease activity, facilitates the autocleavage of phage repressor [25], allowing initiation of transcription from the early *P*_L_ and *P*_R_ promoters (Fig. 1). Transcription from *P*_L_ results in expression of N protein, which acts to modify RNA polymerase initiating specifically at *P*_L_ and *P*_R_ to a form resistant to downstream terminators [26]. N-modified transcription from *P*_R_ transcends downstream terminators resulting in Q expression. Q in turn modifies transcription initiating at the late *P*_R_′ promoter to a termination-resistant form allowing expression of downstream genes [27], including *stx A* and *B* in *stx*-phages [14], [17], [18], [28].

A λ prophage fails to induce if the repressor gene (*cI*) has a mutation that inhibits autocleavage [29], [30]. These mutations, called *ind*, change amino acid residues within the repressor that participate in a serine protease activity that catalyzes autocleavage [25].

We have previously suggested that the induced subpopulation is responsible for Stx production and release [14]. Lysogens with most lambdoid prophages are stable with only an extremely small fraction of the population, in the absence of an external inducing agent, sustaining sufficient DNA damage to be induced, a stochastic process referred to as “spontaneous induction” [25]. It has been suggested that collapse of the replisome in normally growing bacteria caused by single-stranded breaks or noncoding lesions may be an internal event responsible for spontaneous induction [31]. DNA damage-inducing agents change induction from a stochastic to a deterministic process that activates RecA and, in turn, repressor cleavage [32]. Although *recA* mutants have been used to study conditions where the prophage fails to be induced and Stx is not expressed [33], such an experimental approach suffers from the disadvantage of the pleiotropic effects on bacterial physiology due to loss of RecA activity [34], [35]. Using a phage with an *ind* mutation avoids this problem by limiting the failure of SOS control only to the prophage with the *ind* mutation.

Linkage of Stx expression to prophage induction raises the question as to whether the intestinal environment increases Stx levels by causing prophage induction. One way this could occur would be by increasing DNA damage in the bacterium. In vitro experiments showed a modest increase in Stx production by an O157:H7 strain when bacteria were cultured with neutrophils [36], which produce H_2_O_2_ that can cause DNA damage leading to an SOS response and prophage induction.

Here, we report experiments with the O157:H7 strain EDL933 and derivatives of EDL933 that carry a 933W prophage with a *cI ind* mutation. Using a germ-free mouse model of disease, we show that whereas the parent EDL933 with wild-type 933W prophage produces high levels of Stx in vivo and causes severe disease that can lead to death, a derivative isogenic except for a *cI ind* mutation in the 933W prophage produces extremely low levels of Stx2 and does not cause any observable disease. These results provide compelling evidence that induction of the 933W prophage is a major factor in pathogenesis of EDL933 and prophage induction may play a role in the severity of infection by other O157:H7 strains. Using an EDL933 SIVET reporter strain, which survives induction but undergoes a change in antibiotic resistances following induction, we show that the intestinal environment contributes to induction of the 933W prophage in EDL933.

## Results

### Characterization of the 933W *ind* mutant in EDL933

Induction of lambdoid prophages, including that of 933W, occurs when the repressor protein autocleaves in the presence of activated RecA protein [25]. Mutations, *ind*, in the *cI* gene result in a noncleavable repressor and thus an uninducible prophage [29]. The strategy used to obtain the *ind* mutation in the *cI* gene of the 933W prophage in EDL933, a change of Lys codon 178 [suggested by John Little [37]], was based in part on the procedure previously employed by our laboratory to construct an identical point mutation in the *cI* gene of the 933W prophage in strain K9675 [19]. Sequencing confirmed that the *cI* gene in EDL933 with the mutant 933W had only the designed nucleotide substitution at codon 178. The mutation, named *ind1*, is a change of the Lys codon to an Asn codon (K178N). This change interferes with the autocatalytic serine protease activity of the CI repressor [38], rendering the prophage uninducible. We will refer to the derivative of EDL933 carrying the 933W prophage with the *cIind1* mutation as EDL933*cIind1* (Table 1). This strain carries the *stx2* genes and differs from EDL933 only by the 933W *cI* mutation.

**Table 1:** Strain and plasmid list.

Strain Name	Relevant Information	Source/Reference
K37	Nonpathogenic laboratory *E. coli* strain derived from NIH strain N99	[72]
K9675	K37 (933W)	[19]
DY406	W3110 λ*cI857* Δ(*cro-bioA*) *N-kil::cat-sacB*	[73]
EDL933	Isolated from ground beef, Serotype O157:H7	[74], [75]
K10595	K37 (933W*cIind* (K178N)	[19]
DY378	W3110 λ*cI857* Δ(*cro-bioA*)	[64]
K9685	DY406 (*cat-sacB* (CSB))	Court lab
K10373	DY378 *cat*	[76]
K10985	EDL933 pKD46-*ampR*	This work
K11078	EDL933 (933W *N*::*kan*) pKD46-*spc*	This work
K11084	K11078 (*cro*::*tnpR 168*, *OP*::*amp*)	This work
K11114	11084 (CP-933V *N-cII*::*cat-sacB*)	This work
K11115	K1114 (CP-933V Δ*N-cII*)	This work
K11161	K1115 *lacZ::cat*	This work
K11173	EDL933 SIVET 1: (*cat::resC-tetR-resC::cat*)	This work
K11349	EDL933 (933W*cIind*1)	This work
K11604	EDL933 SIVET 2: (*cat::resC-kanR-resC::cat*)	This work
K11607	K11604 (933W*cIind*1 KanR)	This work
K11608	K11604 (933W*cIind*1 CamR)	This work
Plasmid	pJLTnpRhygro	This laboratory
Plasmid	pKD46 (ampicillin R)	[65]
Plasmid	pKD46spcR (spectinomycin R)	This work
Plasmid	pKD46hygR (hygromycin R)	This work

**Figure 2:**
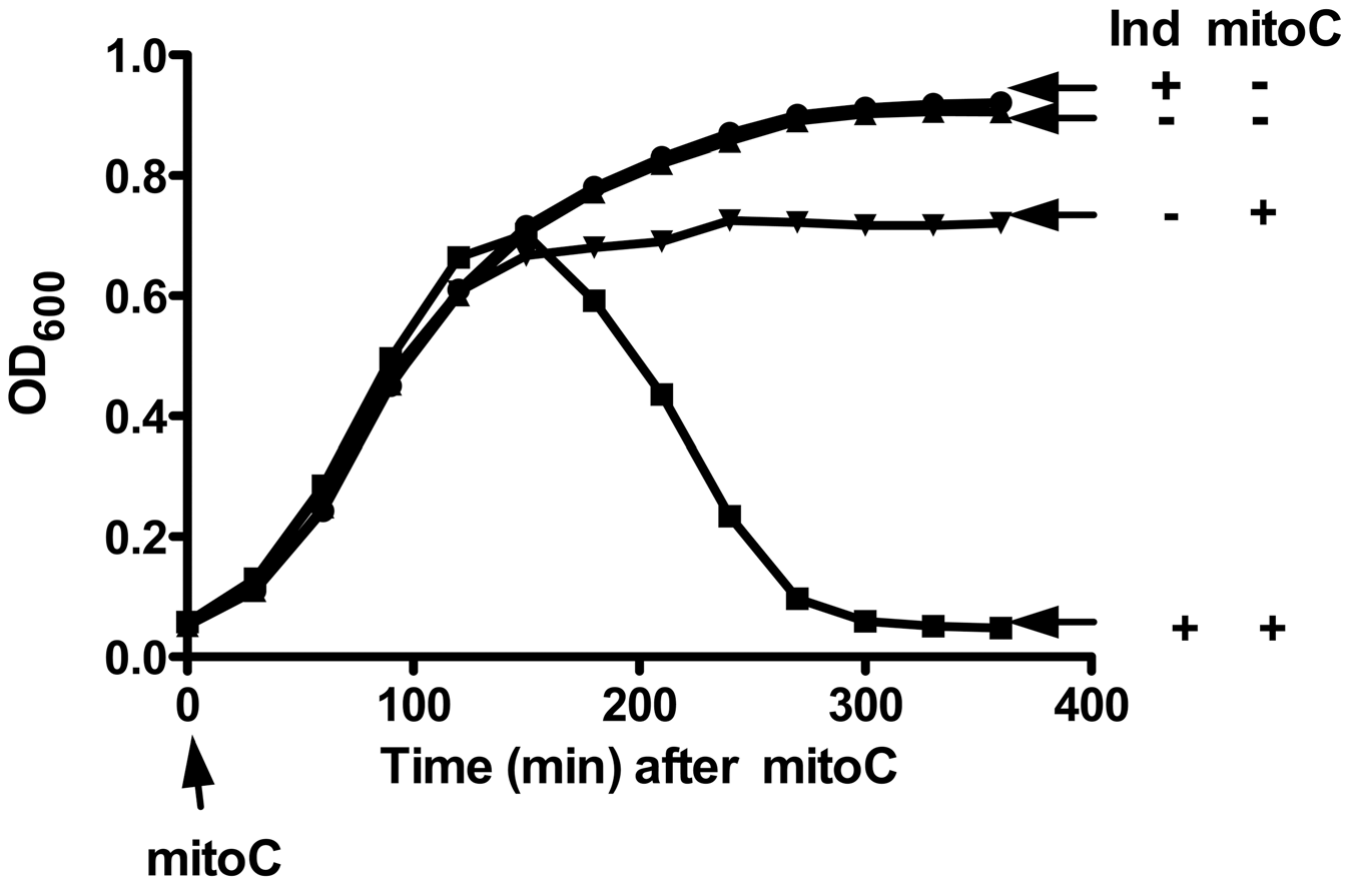
Induction and lysis following mytomycin C treatment. Aliquots of EDL933 and the *cIind1* derivative untreated and treated with 2 µg/ml of mitomycin C were incubated with shaking at 37°. Ind: (+) EDL933, inducible, (−) EDL933*cIind1* (K11349), not inducible. Mitomycin C: (+) treated, (−) untreated. Vertical arrow indicates time mitomycin C added.

To assess the effectiveness of the *ind1* mutation on prophage induction, we treated EDL933 and EDL933*cIind1* with mitomycin C [39]. At an appropriate concentration, this DNA damaging agent activates the SOS response of most of the population sufficiently to induce the prophage [40]. Treatment of the EDL933 parent with 2 µg/ml of mitomycin C led to full induction of the culture; i.e., lysis was nearly complete (Fig. 2). Identical treatment of EDL933*cIind1* failed to cause lysis (Fig. 2). This result confirms that the *ind1* mutation blocks induction of 933W. Additionally, it shows that the inducing agent does not cause any of the large number of defective prophage in EDL933 [41] to express lytic activity. This finding provides direct evidence that induction of 933W is not only responsible for Stx2 production, as shown below, but also for the lysis that releases Stx2 from the bacterium.

### Shiga toxin production

**Figure 3:**
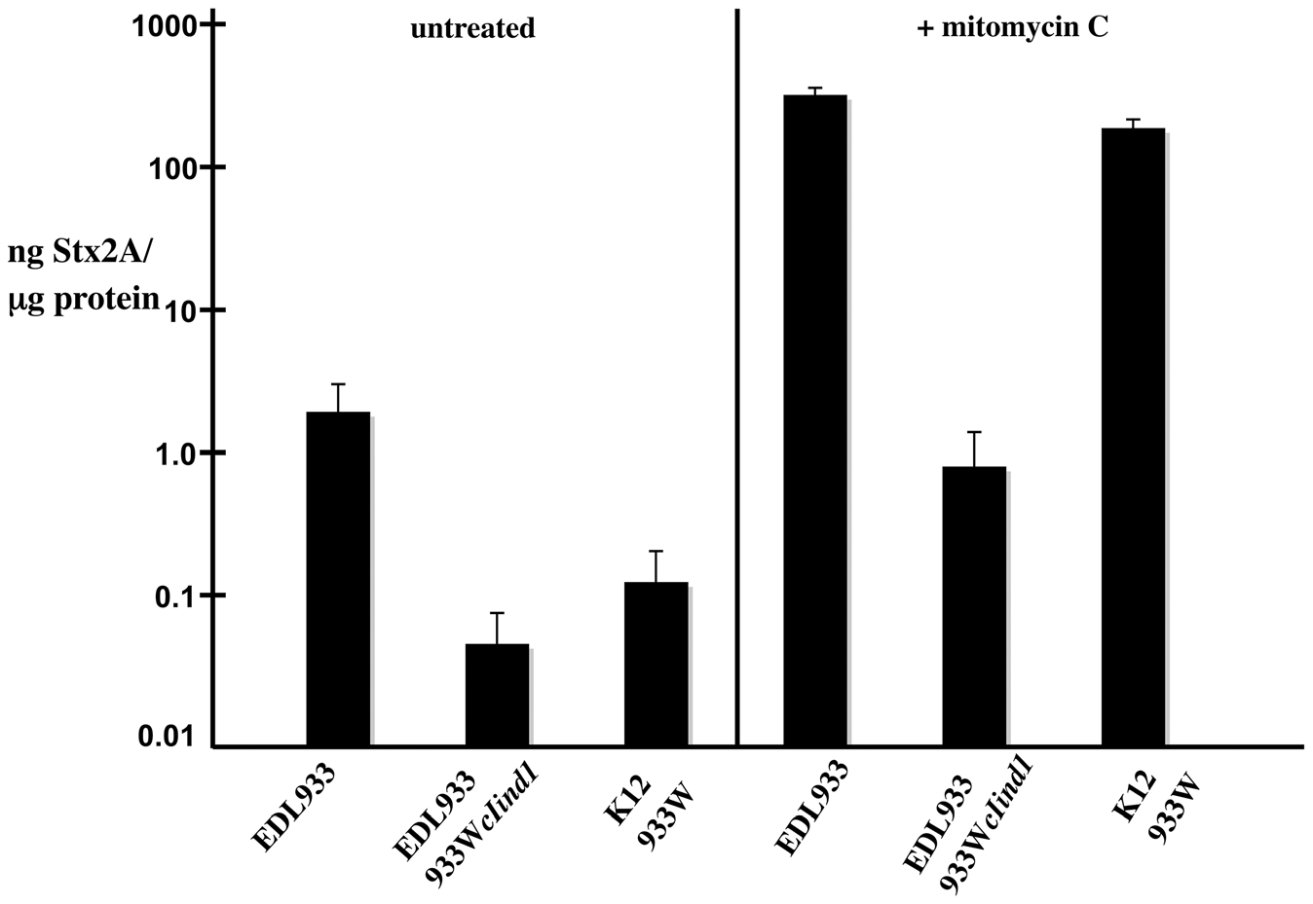
Expression of Stx. Levels of Stx were determined as outlined in Materials and Methods. Mitomycin C was used at a concentration of 2 µg/ml. Strains: EDL933, EDL933*cIind1* (K11349), and K37-933W (K9675). Error bars indicate standard deviations. *P* values (mc = mitomycin C treated): (1) EDL933×EDL933 (mc) = 6.6×10^−7^; (2) EDL933×K11349 = 0.0056; (3) K11349×K11349 (mc) = 0.018; (4) EDL933×K9675 = 0.0069; (5) K9675×K9675 (mc) = 3.5×10^−6^; (6) EDL933 (mc)×K9675 (mc) = 0.00044.

We used an ELISA to assess Stx2A levels; comparing levels in EDL933 with those in the EDL933*cIind1* derivative and the nonpathogenic 933W lysogen K9675. In the absence of an inducing agent, the parent EDL933 expresses ∼40 times the level of Stx2 expressed by EDL933 *cIind1* mutation (Fig. 3). This result provides compelling evidence that in culture a significant fraction of Stx2 production derives from the subpopulation of EDL933 in which the 933W prophage is induced. These results are only partially consistent with our previous findings with strain K9675 [19]. In that study we found that in the absence of an external inducing agent, the level of Stx2A produced by K9675 was ∼10 fold lower than the level produced under these conditions by EDL933 with its wild-type 933W prophage. In the current study, we confirmed these findings, showing that in the absence of an external inducer (spontaneous induction) EDL933 produces ∼10 times more Stx2A than K9675 (Fig. 3).

To rule out the possibility that the low Stx2A levels in the nonpathogenic strain resulted from alteration of the prophage or the host, we measured Stx2A production from another nonpathogenic K12 related strain, MC1000 [42], with a 933W prophage. As above, we observed the lower level of Stx2A expression in the non-pathogenic strain compared to EDL933 (data not shown). Although comparison of spontaneous induction shows that EDL933 produces ∼10 fold higher levels of Stx2A than K9675, the source of Stx2A for each is primarily that fraction of the population in which the prophage is induced (this study and Tyler et al. [19].

### Prophage induction and Stx2 production

To specifically assess the role of induction of the 933W prophage in Stx2 production, we determined Stx2 levels following treatment with mitomycin C (2 µg/ml). As shown in Fig. 3, mitomycin C treatment resulted in a 100 to 200-fold increase in Stx2 production by EDL933. Although K9675 produced significantly less Stx2 than EDL933 in the absence of an inducing agent, it produced about the same levels of Stx2 following treatment with mitomycin C as similarly treated EDL933.

The EDL933*cIind1*culture treated with mitomycin C produced 5- to 10-fold more Stx2 than the untreated culture. Although two orders of magnitude lower than the Stx2 production reached by EDL933 treated with mitomycin C, the increased levels we observed with the treated EDL933*cIind1* were reproducible. The increase in Stx2 following mitomycin C treatment is consistent with the observation of measurable levels of Stx2 produced by EDL933*cIind* in the absence of an inducing agent. Either the mutant repressor retains some ability to autocleave (leaky mutant) in the EDL933 environment or there is an alternative route to Stx2 expression. However, in either event the production of Stx2 is extremely low in the presence of the *cIind1* mutation.

### Prophage induction and EDL933 pathogenicity

Results of clinical studies of children with EHEC infection show that phage induction likely plays an important role in the disease; e.g., those treated with antibiotics that elicit an SOS response may experience more severe outcomes [21]. In mice, treatment with ciprofloxacin (an antibiotic that elicits the SOS response) also results in greater in vivo expression of Stx, likely via prophage induction [43].

Although suggestive, these findings are far from definitive. As discussed, the SOS response has pleiotropic effects on bacterial gene expression and does far more to affect cell physiology than induce prophage [34], [35]. Relevant to our studies, treatment of EDL933 with the DNA damaging agent norfloxacin results in changes in the expression of a number of prophage and non-prophage genes in EDL933 [44]. Because the only effect of the *ind1* mutation is to interfere with induction of the 933W prophage, experiments with EDL933*cIind1* allowed us to ask specifically how significant induction of the 933W prophage is in causing the pathology associated with EDL933 infection.

**Figure 4:**
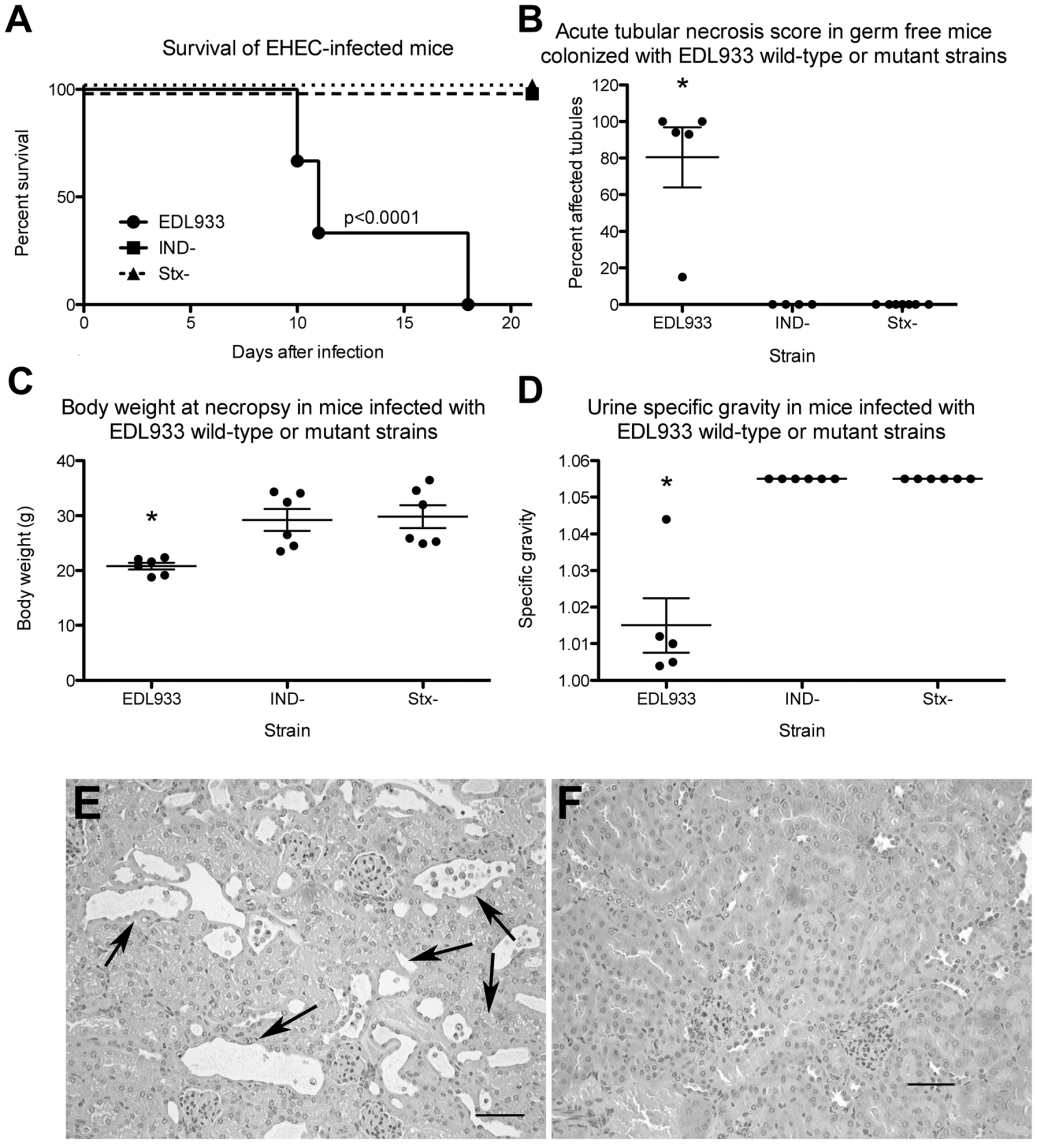
Disease in mice infected with EDL933. A: Kaplan-Meier survival curve; B: Acute tubular necrosis score; C: Body weight at necropsy; D: Urine specific gravity; E: Hematoxylin and Eosin stained section of kidney from a mouse inoculated with EDL933. Many tubules are necrotic and contain cellular debris (arrows). A few glomeruli contain fibrin thrombi (arrowheads). F. Mouse infected with the EDL933*cIind1* negative mutant. Tubules and glomeruli are normal. Bars = 50 microns. * Significantly different from mice infected with Ind- or Stx- mutants, p<0.001. Slides were scored blind without knowledge of their source. Animals were euthanized and tissues removed for examination; at 10 days post infection with EDL933 (day at which they became moribund) and at 21 days post infection with EDL933*cIind1I* (did not become moribund).

The germ-free mouse has proven an effective and practical animal model for studying the pathology of EHEC infection [7]. We found that germ free mice infected with O157:H7 strains such as EDL933 develop acute renal tubular necrosis and renal glomerular thrombosis leading to renal failure and death. In the same study, we also reported that a similar infection with a derivative of EDL933 isogenic except for a deletion of the *stx2* genes does not result in any of the pathogical changes seen with the wild-type parent strain. Hence, in this animal model, all of the described pathological changes result from the action of Stx2. For these reasons, we chose the germ-free mouse to assess the role in the disease process of induction specifically of the 933W prophage carried by EDL933. Groups of 6 (3 female and 3 male) germ-free Swiss-Webster mice were used in the experiments. They were infected with one of three bacteria, EDL933 or either of two isogenic strains that differed by having the *Δstx::cat* deletion substitution or the *cIind1* point mutation. For all strains tested, each animal was challenged with 10^6^ cfu administered orally. All three groups of mice were equally colonized over the seven days of the experiment in which bacteria in the feces were measured (∼10^10^ cfu/g). As expected from our previous work, all 6 mice infected with the wild-type EDL933 parent strain became moribund prior to the scheduled time mice were euthanized at three weeks. All mice infected with the *Δstx::cat* deletion derivative showed no signs of disease. Like the latter group, mice infected with EDL933*cIind1* showed no signs of disease (Fig. 4).

Figure 4A shows a Kaplan-Meier survival curve of mice inoculated with the three strains. All 6 mice given EDL933 became moribund or died prior to 21 days after inoculation. At necropsy, these mice were dehydrated and thin, and their ceca were distended with fluid contents. Mice in this group had moderate-severe acute renal tubular necrosis (Fig. 4B), failed to gain weight as indicated by significantly lower body weights at necropsy (Fig. 4C), and all and dilute urine (Fig. 4D), indicating renal failure. Histologically, renal disease was characterized by necrosis of renal tubules and occasional glomerular fibrin thrombi (Fig. 4E). Mice in the other two groups did not show any signs of disease, and had normal renal morphology Fig. 4F). As noted above, cecal colonization was similar in all three groups of mice ruling out poor colonization as an explanation for the failure of EDL933*cIind1* to cause disease.

As discussed, in vitro EDL933*cIind1*produces measurable levels of Stx2, raising the question of whether it produces measurable levels of Stx2 in the infected mouse. Although there was wide variation, we found low but measurable levels of Stx2 in the feces of some of the mice infected with EDL933*cIind1*, 0–300 ng/ml of feces. Much higher levels of Stx2, with considerable variation, were found in the feces of mice infected with EDL933, 6529±4432 ng/ml of feces (P = 0.0039).

### Effect of in vivo environment on prophage induction

Based on the RIVET (recombinase based in vivo expression technology) [45], [46], we developed SIVET (selectable in vivo expression technology), with the aim of determining if there is any effect on prophage induction when bacteria are in the intestine. Studies with the first generation SIVET, constructed in the nonpathogenic *E. coli* strain MC1000, established this reporter system as a valid method for measuring prophage induction [47]. Here we report construction of a second generation SIVET through modification of EDL933 (see Materials and Methods for details). Figure 1-II outlines the essential features of the SIVET system. Briefly, the 933W and 933V prophages in EDL933 were genetically altered so that functions lethal to the bacterial host [48] are not expressed upon induction and the bacterium therefore survives challenge with an inducing agent. The *tnpR* gene from the γδ transposon [49] was cloned downstream of the 933W early *P*_R_ promoter distal to the *cro* gene. Thus, following induction of the 933W prophage transcription initiating at the phage promoter *P*_R_ results in production of the TnpR resolvase that, in turn, acts at another site on the bacterial chromosome to excise a *kanR* cassette that interrupts a *cat* gene. This recombination serves two purposes, establishes a functional *cat* gene and removes the *kanR* cassette, conferring CamR. Hence, upon induction of the altered 933W prophage there is an irreversible and inheritable change of the host bacterium from KanR/CamS to KanS/CamR. The fraction of the total bacterial count that is CamR provides a measurement of the number of bacteria in which the prophage was induced.

**Figure 5:**
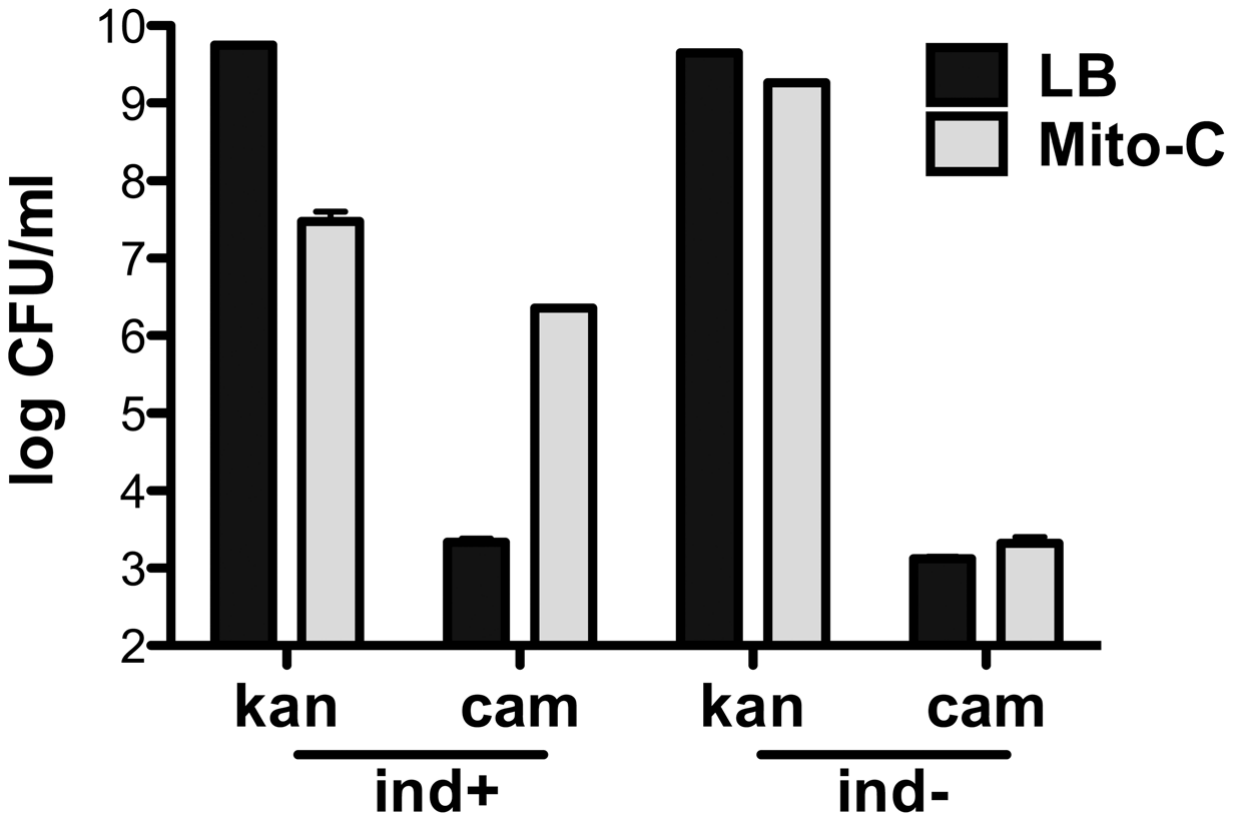
SIVET induction by treatment with mitomycin C. SIVET strains EDL933 (K11604) and EDL933*cIind1* (K11607) were treated as described in the Materials and Methods. Bars show colony forming units with and without mitomycin C treatment; Left (*ind*+) EDL933 and right (*ind*−) EDL933*cIind1*. Error bars represent standard error of the mean.

That this change is due to prophage induction is shown by the results of the following experiments. First, treatment of the SIVET strain with mitomycin C, known to cause prophage induction [39], results in an increase of ∼1000 fold in CamR colonies and a reduction of ∼1000 fold of KanR colonies (Fig. 5). Second, treatment of a *cIind1* mutant derivative of the SIVET strain (K11607) under exactly the same conditions used with the SIVET parent failed to cause any measurable change in the levels of KanR or CamR bacteria (Fig. 5).

In the following in vitro and in vivo experiments, the ratio of CamR/KanR SIVET was standardized to simplify the presentation using what will be referred to as the “Induction Index”. This function is calculated as the log_10_ of (CamR/KanR output)/(CamR/KanR input) (for details see Materials and Methods). Because of the way the Induction Index is calculated, the starting point in the graphs, the input, is equal to log_10_ (1) or 0. This allows changes in induction to be monitored by observing movement of the Index away from 0.

**Figure 6:**
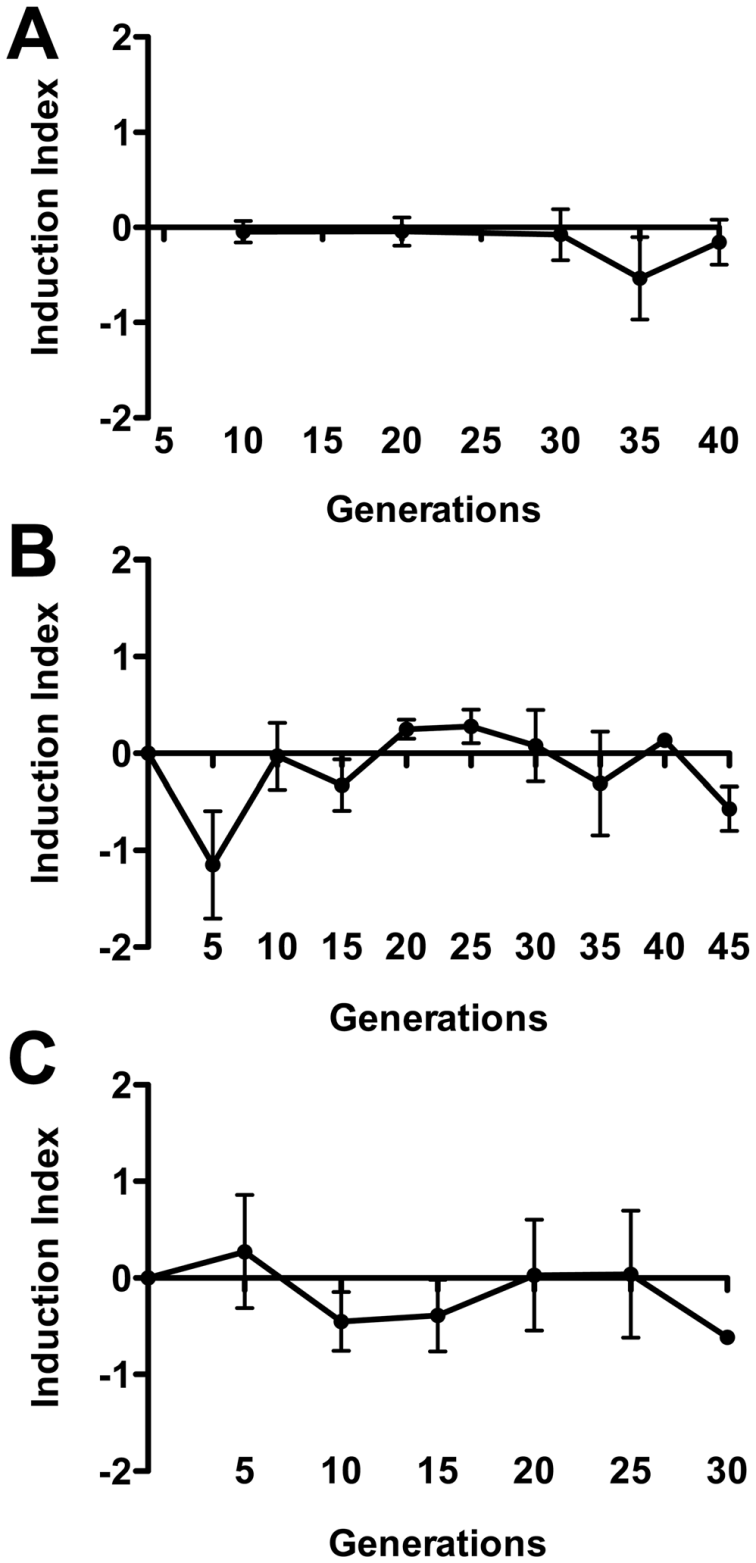
Measurement of in vitro prophage induction. SIVET strains were grown in LB broth and serially diluted into fresh LB. Cultures were grown to desired densities and concentrations of CamR and KanR bacteria in cultures measured at indicated points in growth cycle. Multiple rounds of dilutions and growth allowed for indicated rounds of duplication. Where appropriate, low dilutions ensured that any CamR bacteria would be carried over during dilutions. Graphs show ratios of CamR/KanR on a log scale (calculated using the Induction Index formula) over the indicated number of generations. A. SIVET *cIind1*strains K11607 (locked in KanR form) and K11608 (CamR) were grown together in LB to stationary phase at which time bacterial counts were determined and dilutions were made for next round of growth. Error bars represent standard error of the mean. B. Cultures of K11604 were grown to stationary phase at which time bacterial counts were determined and dilutions were made for next round of growth. C. Cultures of K11604 were grown to late log phase at which time dilutions were made for next round of growth. Aliquots were removed at mid-log phase for determination of bacterial counts.

The only way we see the ratio deviate, beyond expected scatter, from 0 on the Induction Index, is if one of the two populations increases more than the other either by a growth advantage or by addition of newly generated derivatives. To rule out alteration in the induction index due to a growth advantage of one or the other marked strain, we used two SIVET derivatives; one, K11607, locked in the KanR form by virtue of the *cIind1*mutation and the other, K11608, a derivative of K11607 which is isogenic except for the excision of the KanR cassette and thus is locked in the CamR form. The CamR/KanR ratio (calculated employing the formula used to generate the Induction Index) following coinfection with the locked in CamR and KanR derivatives hovers around 0 (Fig. 6A). Since there is no growth advantage to either form, any positive increase in the CamR/KanR Induction Index of the parental SIVET would have to be explained as addition by conversion from the KanR population to the CamR population, a direct consequence of induction of the 933W prophage in the KanR bacteria.

As discussed above, a small fraction of a population of lysogens growing in the absence of an added inducing agent undergo induction, a process called spontaneous induction [25]. To determine whether spontaneous induction of the SIVET prophage adds to the population of CamR bacteria, we measured the CamR/KanR ratio, determined as the Induction Index, over the course of a large number of doublings in vitro in two different ways (Fig. 6). In both approaches, the SIVET strain was serially passaged in vitro for a number of generations in LB medium and the CamR and KanR populations periodically measured by viable counts. In one set of experiments, the SIVET bacteria were grown to stationary phase and diluted 10-fold for the next passage (Fig. 6B) while in the other, the bacteria were kept in log phase and diluted from an OD_600_ of ∼1.0 to an OD_600_ of 0.1 for the next passage (Fig. 6C). Both protocols yielded similar experimental results; the Induction Index remained relatively constant over many doublings, hovering around 0. These results lead us to conclude that spontaneous induction does not significantly affect the CamR/KanR ratio. We consider these results further in the Discussion.

### Induction in the mouse intestine

**Figure 7:**
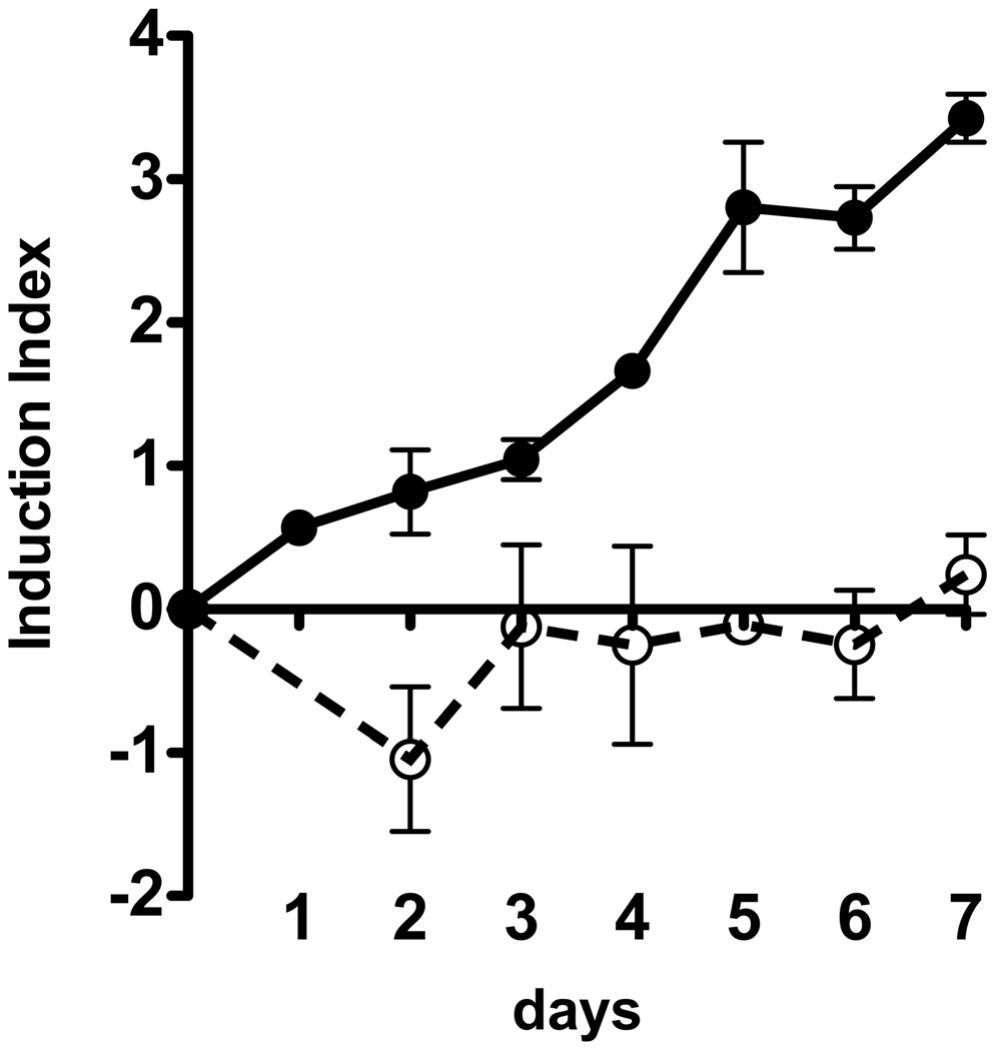
Measurement of in vivo prophage induction. Germ-free mice were infected with 10^6^ bacteria. At the indicated times feces were collected, suspended in diluent, and dilutions plated on LB plates, with kanamycin (30 µg/ml), or chloramphenicol (9–10 µg/ml). Graphs show ratios of CamR/KanR on a log scale (calculated using the Induction Index formula) over the indicated number of days. (•) SIVET strain K11604. (○) K11604 derivatives with 933W *cIind* mutation; K11607 (locked in KanR) and K11608 (CamR) were co-inoculated. Error bars represent standard error of the mean.

To determine if the intestine environment contributes to prophage induction and thus Stx production, we employed the ELD933 SIVET strain using the infection protocol as described above. Each mouse was orally infected with ∼10^6^ SIVET bacterium. Because the 933W prophage was mutationally disarmed (see Materials and Methods for details) and thus does not produce Stx2, as expected, mice infected with EDL933 SIVET did not show signs of disease. Feces were isolated each day for seven days and bacterial counts were determined by plating on LB agar plates containing kanamycin or chloramphenicol. The total EDL933 SIVET count remained relatively constant over the course of the experiment, ∼10^8^ CFU/g of feces, although slightly decreasing by the seventh day (data not shown). The Induction Indexes over the 7 days presented in Fig. 7 were compiled from results of three independent experiments, each comprised of five mice.

By day seven the Induction Index has increased by over three logs. The study was terminated at day 7, when the onset of severe disease caused by EDL933 usually occurs [7].

To determine if the change in the CamR/KanR Induction Index during in vivo growth of SIVET reflects a difference in viability of the two forms of the SIVET, we employed the SIVET pair K11607 and K11608. These derivatives, as discussed above, are locked in either the KanR or CamR form. Mice were co-infected with K11607 and K11608 and followed essentially as described for the in vivo SIVET study outlined above. Examination of fecal samples showed that the ratio of CamR/KanR (calculated using Induction Index formula) did not significantly change over the course of 7 days (Fig. 7); i.e., neither form of SIVET has a growth advantage during in vivo growth. Hence, the null hypothesis stands and we conclude that the increase of the CamR/KanR Induction Index observed during growth in the mouse intestine results from prophage induction.

Based on this collection of data, we conclude that there is significant induction of the 933W prophage in the germ free mouse intestine. Since Stx2 production is directly linked to 933W induction, it follows that the intestine, through action of a yet to be identified factor(s), stimulates Stx2 production through induction of the 933W prophage.

## Discussion

With the information gained from sequencing numerous bacterial genomes, it has become apparent that virulence factors are commonly located in genomes of prophage [50], [51]. Introduction of a new function, such as a virulence factor, to a bacterium by a prophage is referred to as lysogenic conversion. Although Stx2 is an example of a phage-encoded toxin whose expression is controlled by the phage regulatory cascade, many other phage-encoded toxins are expressed independently of prophage regulatory functions. This is true for the classic toxin of *Corynebacterium diphtheriae* [52] and of cholerae toxin (CTX), which is encoded in the genome of the CTXΦ prophage [53]. Expression of CTX is controlled by a complex circuitry of proteins encoded by regulatory genes located outside of the prophage genome [54]. Observations like these led to the idea that phage, like other mobile elements, serve as agents that can transfer genetic information from one bacterium to another [55]. However, at least in the case of *stx*-phages, the phage serves a wider role, not only being the source of transfer, but also the regulator of expression from the transferred virulence gene [16].

The construction of a derivative of EDL933 with the *ind1* mutation in the 933W prophage coupled with an animal model that mimics, to a large degree, the human disease, has allowed us to specifically assess the contribution of induction of the 933W prophage to the disease process. Like its EDL933 parent, EDL933*cIind1* effectively colonizes the host intestines. However, unlike the parental strain, the *ind1* strain fails to elicit any of the hallmarks of an EHEC infection; e.g., physical signs of illness, renal disease, and death. That EDL933*cIind1* colonizes the host intestine is consistent with our previously reported findings showing that a derivative of EDL933 with a deletion-substitution of the *stx2* genes colonized as well as the parent strain with a functional *stx2* gene [7]. This observation is contrary to the findings of Robinson et al. [56], who reported that colonization was reduced if the O157:H7 strain did not express Stx2. As we have suggested previously [7], this difference may reflect our use of germ-free mice, while Robinson et al. used mice with normal microbiota.

Our results provide evidence that the major pathogenic effect of EDL933 results from induction of the 933W prophage. Hence, the phage regulatory cascade plays a central role in the pathogenesis of this O157:H7 strain and likely many others. Since repressor auto-cleavage requires activated RecA protein, which, in turn, is a product of the SOS response, it is primarily that subpopulation of bacteria, with a sufficiently vigorous SOS response that induces the 933W prophage and results in the production and release of Stx2.

Our observation that Stx2 production and disease in the mouse are directly related to induction of the 933W prophage raises the question as to whether there is a factor(s) in the intestines that increases the SOS response resulting in increased prophage induction beyond that expected from results of in vitro experiments. Such a role was found for a factor in human pharyngeal cells that induces a group A Streptococcus prophage [57]. And a small but significant level of induction of Stx was observed when an EHEC strain was co-cultured with human neutrophils [36]. In a similar manner, a factor(s) in the intestines that induces an SOS response might increase the levels of Stx produced by a population of infecting EHEC. Such a factor(s) could be a product of the host (e.g., neutrophils). Not considered here is the possible importance of the interaction between the microbiota and the mammalian intestine in the SOS response and resulting Stx2 production [58]. Our studies with germ free mice show that even in the absence of the normal microbiota there is sufficient prophage induction to produce and release levels of Stx capable of causing renal disease and death.

Constructed regulatory networks as biosensors have wide biological applications [59]. The studies reported here demonstrate the utility of the comparatively simple SIVET regulatory network as a tool for identifying conditions where prophage induction is enhanced. First, treatment in vitro of SIVET with the inducing agent mitomycin C results in overwhelming conversion of KanR to CamR (Fig. 5), confirming that SIVET responds to inducing agents as designed. Second, the experiments with the 933W *cIind* SIVET derivatives showed that the increase in CamR relative to KanR colonies observed during in vitro and in vivo growth is not due to a growth advantage of the CamR variants (Figs. 6 and 7). Third, no significant change in the ratio of CamR to KanR was observed over a large number of doublings during continuous in vitro growth of SIVET in the absence of an inducing agent (Fig. 6). This observation held true whether cultures prior to dilution were allowed to grow to stationary phase or were maintained in log phase. In each case dilutions were at a sufficiently high level to ensure that CamR bacteria were carried over during each dilution. It might be expected that CamR bacteria contributed de novo by induction should add to the growing population, resulting in an increase in the CamR/KanR ratio. However, the in vitro experiments failed to show an increase in the Induction Index over a large number of doublings (we discuss this apparent paradoxical finding in detail below). Based on these results, we conclude that spontaneous induction (induction in the absence of a known inducing agent) of the 933W prophage fails to lead to a measurable increase in conversion of SIVET from KanR to CamR. Hence, SIVET is not sufficiently sensitive to distinguish no induction from low levels of induction.

By eliminating obvious alternative explanations and showing that mitomycin C treatment results in an increase in the SIVET CamR/KanR ratio, these results confirm that SIVET can be used to identify the presence of inducing agents. Moreover, the failure to observe changes in the Induction Index over many rounds of doubling during in vitro growth, in the absence of an extrinsic inducing agent, indicates that even small measurable increases in the Induction Index should provide evidence of an extrinsic inducing agent.

In the light of this background information, the >3 log increase in the Induction Index observed in SIVET isolated from feces (Fig. 7) over the seven days following the initial infection provides evidence for action of an inducing factor in the mouse intestinal tract. We suggest three alternative, but not mutually exclusive, scenarios to explain this increase in the rate of induction: 1) a substantial portion of the bacteria reach a section of the intestinal tract that contains resident inducing activity; 2) the infection causes an increase in the amount and/or activity of a resident inducing activity; or 3) infection attracts an inducing activity or a cell (e.g., neutrophils) producing an activity. Since Stx2 production, in large measure, is directly related to phage induction (Fig. 3), the intestinal environment likely contributes to the severity of the EHEC infection.

Although we failed to observe any significant change in the Induction Index over many generations of in vitro growth, an increase in the Induction Index over time might be expected because spontaneous prophage induction [25] should result in TnpR expression and, at some level, conversion of KanR to CamR bacteria. This, in turn, would add to the total of CamR population over the number produced by replication of preexisting CamR population resulting in an increase in the CamR/KanR ratio.

We used mathematical modeling to gain a quantitative understanding of what the expected Induction Index over time would look like if all of the spontaneously induced KanR bacteria were able to contribute immediately to the CamR bacterial population. Based on a starting Induction Index of 0, the model adds the newly produced CamR bacterium at each division to the growing preexisting CamR population, predicting an increase in the Induction Index over time as shown in Fig. S1. If we assume a doubling every hour over the seven days of in vivo growth, the model predicts the Induction Index would increase a little over one log and, even assuming a doubling time of 20 minutes, the Index would increase by slightly over two logs, both substantially less than the over three logs observed in the in vivo SIVET experiment.

The counterbalancing actions that we see as potentially reducing the contribution of spontaneous induction might make to the CamR population, include: 1) as discussed above, SIVET may not be sufficiently sensitive to distinguish no induction from low induction; 2) there may be a delay in initiation of growth following recovery from the consequences of DNA damage that caused the induction [60], [61]; i.e., a phenotypic lag (graphed in Fig. S1); 3) removal of the KanR cassette may occur in only one of the multiple bacterial chromosomes [62] resulting in segregation of both CamR and KanR derivatives from a single induced KanR bacterium and thus resulting in no change in the CamR/KanR ratio ; and 4) there may be sufficient DNA damage in some of the bacteria to block further growth, compromising survival of those bacteria. This subpopulation would be part of the induced pool that although theoretically adding to the CamR population would not be alive to do so. Although collectively these actions could explain our results, we are far from having a definitive answer as to how the Induction Index maintains this steady state. Nor can we explain how the ratio of CamR/KanR colonies reaches a steady state that is maintained for many generations. However, failure of SIVET to identify low level induction (spontaneous), but identify high level induction, as with mitomycin C, indicates measurements by SIVET are likely to be an under representation.

## Materials and Methods

### Ethics statement

All animal protocols were approved by the University Committee on Use and Care of Animals at the University of Michigan Medical School. The University of Michigan is fully accredited by the Association for Assessment and Accreditation of Laboratory Animal Care, International (AAALAC, Intl) and the animal care and use program conforms to the standards of “The Guide for the Care and Use of Laboratory Animals” (published by the NRC).

### Bacteria, phage, and plasmids

See Table 1.

### Primers and oligonucleotides

See Table 2.

**Table 2:** Primers and oligonucleotides.

#	Name	Oligonucleotides	Role
**1**	5′*N::kan*	5′–GGTTTGCTGCCTAATTTCATTTTCTGGCGACCAACACAAGAGCGTAATGCTCTGCCAGTG–3′	Construct 933W *N::Kan*
**2**	3′*N::kan*	5′–TCCCACCTACCACACCACCAAAGTTCATCAGGAGGTCTATAGGAATTCCCCGGATCCGTC–3′	Construct 933W *N::Kan*
**3**	5′ *catsacB* (inactivate CP933V)	5′–TTCTCGCTGTGTTGGCTTGCTGTAGCTTGCTTGTGCCAGTTACTTAGATATTGGCCTTGG–3′	CP933V *NcII*
**4**	3′ *catsacB* (inactivate CP933V)	5′–TGCCGTACCTTTGGATTCTTTCCAGACAATGGTTACACTGTCCATATGCACAGATG–3′	CP933V *NcII*
**5**	5′ *cat* (synthesize *cat::resCtetresC::cat* cassette)	5′–ATGACTATGATTACAGATTCACTGGCCGTCGTTTTACAAATGAGACGTTGATCGGCACG–3′	*lacZ::cat* construct
**6**	3′ *cat* (synthesize *cat::resCtetresC::cat* cassette)	5′–TTATTTTTGACACCAGACCAACTGGTAATGGTAGCGACCAAAAATTACGCCCCGCCCTG–3′	*lacZ::cat* construct
**7**	5′ 933WOR (synthesize *tnpRampR* oligo)	5′–GGCTATAGCCATTCCCCTAC–3′	replaces 933W *cIIOP*
**8**	3′*ren*seq (synthesize *tnpRampR* oligo)	5′–GCTCAGTGATGTAGATGGTC–3′	replaces 933W *cIIOP*
**9**	5′*lac:cat*	5′–ATGACTATGATTACAGATTCACTGGCCGTCGTTTTACAAATGAGACGTTGATCGGCACG–3′	5′ *cat in lacZ gene*
**10**	3′ *lac:cat*	5′–TTATTTTTGACACCAGACCAACTGGTAATGGTAGCGACCAAAAATTACGCCCCGCCCTG–3′	3′ *cat in lacZ gene*
**11**	5′*tetkan*	5′–TTGACAGCTTATCATCGATAAGCTTTAATGCGGTAGTTTATCTATGGACAGCAAGCGAACCGG–3′	replace *teR* with *kanR*
**12**	3′*tetkan*	5′–TCAGGTCGAGGTGGCCCGGCTCCATGCACCGCGACGCAACGCGGGGAGGCAACCCCAGAGTCCCGCTCAG–3′	replace *teR* with *kanR*
**13**	5′*resCkan*	5′–ATTTTTTGTTATAACAGACACTGCTTGTCCGATATTTCATTTAGGATACAAGCGTAATGCTCTGCCAGTG–3′	Synthesize *resCkan*
**14**	3′*resCkan*	5′–ATTAACAGCACTGTTTTTATGTGTGCGATAAttgaTAATATTTCGGACGGAGGAATTCCCCGGATCCGTC–3′	Synthesize *resCkan*
**15**	CP933V Δ*NcII*	5′–TTCTCGCTGTGTTGGCTTGCTGTAGCTTGCTTGTGCGTAACCATTGTCTGGAAAGAATCCAAAGGTACGGCA–3′	replaces *catsacB* in CP933V
**16**	933W*cIind1*	5′–TTTGTCAGAACCGTTGAAGGACACAACATGATTAAcGTTCTTGGCTATGACAGAGATGGAGAATACCAATT–3′	change at *cI* codon 178

### Media

LB, 10 g tryptone, 5 g yeast extract, 5 g NaCl/liter of H_2_O. For LB plates 10 g of agar was included. LB sucrose plates are LB plates without NaCl and made 10% in sucrose. Antibiotics were added at the following concentrations; spectinomycin 80 µg/ml, ampicillin 100 µg/ml (plasmids) 25 µg/ml (chromosomal), kanamycin 30 µg/ml, hygromycin 200 µg/ml, and chloramphenicol 9–10 µg/ml. TB plates, 10 g tryptone, 2.5 g NaCl, and 10 g agar/liter of H_2_O.

### Recombineering

All of our constructs were engineered using the λ Red recombination system, colloquially referred to as recombineering [63]. The λ Red functions were supplied in either of two ways: transiently by a heat pulse freeing a λ promoter on a truncated λ prophage from control by a Ts repressor (*cI857*) so that the downstream *red* genes could be transcribed, using DY378 [64] or from pKD46 and derivatives of that plasmid carrying cloned λ *red* genes by adding arabinose to the growth medium to activate an Ara-regulated promoter [65]. Single-stranded oligonucleotides or double-stranded PCR products of varying lengths having ∼40 nucleotides of flanking sequences with homologies to the target regions were introduced by electroporation into bacteria expressing λ Red functions. The expressed Red functions recombine the introduced DNAs with the target site. In the absence of a selectable marker, a two-step procedure was used: a *cat-sacB* (CSB) cassette [64] was inserted by recombineering and the recombinant selected by resistance to chloramphenicol. This cassette was then exchanged by recombineering with the designed DNA product using as selection resistance to sucrose and confirming by screening for CamS. DNA sequencing by the University of Michigan Sequencing Core Facility confirmed structure of constructs.

### Construction of EDL933 with a 933W *ind1* prophage

Recombineering was used to cross the designed mutation from a single stranded oligonucleotide to the chromosome of strain K10985. The oligonucleotide contained a single nucleotide change that resulted in a replacement of Lys codon 178 (AAG) with an Asn codon (AAC). The following is the sequence of the DNA oligonucleotide (oligo #2) with the mutant nucleotide capitalized: 5′-ccgggtgatgaggtgtttgtcagaaccgttgaaggacacaacatgattaaCgttcttggctatgacagagatggagaataccaatttacaagcattaacca-3′. The pairing of the oligonucleotide with its complementary chromosomal DNA strand forms a C-C mismatch at the position of the nucleotide change. This mispairing is not repaired by the mismatch repair system [66]. In the absence of mismatch repair there is a significant increase in isolation of bacteria with the designed nucleotide change [67]. K10985, an EDL933 derivative with the pKD46 plasmid [65], was prepared for electroporation essentially as described by Murphy and Campellone [68]. Following electroporation, bacteria were resuspended in 10 ml of LB broth and grown at 37°. After ∼5 hrs of growth, dilutions of the bacteria were placed on LB plates and incubated overnight at 37°. The following day colonies were picked and stabbed to an LB plate and a TB plate that was layered with a lawn of K37, a strain that supports growth of 933W. Plates were incubated at 37° for two hours and the seeded plate was UV irradiated (1.6 Joules/M^2^/S for 30 seconds). Following overnight incubation at 37°, a zone of lysis in the lawn showed phage had been synthesized by an induced prophage. Two clones out of 160 tested showed no zones of lysis. These derivatives failed to lyse following treatment with mitomycin C and subsequent DNA sequencing showed that although they both had the *cIind* mutation, only one, EDL933*cIind1*, had no other changes and was selected for further study.

A similar strategy was used to construct an EDL933 SIVET *ind* mutant, K11607, that was KanR. A CamR derivative, K11608, isogenic except for the loss of the KanR cassette and thus converted to CamR, was constructed from K11607 using a plasmid, pJLTnpRhygro, which supplied the TnpR resolvase.

### Toxin assay

Overnight cultures were diluted and grown to early log phase in LB. The cultures were divided into two aliquots; one grown untreated and the other treated with 2 µg/ml mitomycin. Cultures were grown for 3–4 hours, based on time of lysis for the mitomycin C treated culture. Uninduced cultures were diluted every 30 minutes to maintain them in logarithmic growth. Cultures were sonicated 3× for 10 seconds at amplitude of 30% to obtain total cell lysis. Lysates were passed through 0.22 µm filter and concentrated using Amicon Ultra-4 (Millipore). Stx2A levels in supernatants were measured using an enzyme-linked immunoabsorbent assay (ELISA) following a previously published procedure [69] using anti-Stx2A monoclonal and anti-Stx2 polyclonal antisera. Results were determined as ng Stx2A/µg total protein.

### Animal experiments

Germ-free Swiss-Webster mice of both sexes were raised in the University of Michigan Laboratory of Animal Medicine germ free colony, housed in soft-sided bubble isolators, and fed autoclaved water and laboratory chow ad libitum. Inoculations, monitoring of animals, and sample collections were performed as previously described [7]. In brief, mice were inoculated orally with ∼10^6^ cfu of LB-cultured bacteria. Each group of inoculated animals contained 3 male and 3 female mice between 5 and 6 weeks of age. Throughout the experiment and at necropsy, feces or cecal contents were collected for quantitative EHEC culture. Gram stain and aerobic and anaerobic culture were used to demonstrate the absence of microorganisms other than EHEC. Mice remained sterile (except for the infecting EHEC strain) throughout the course of the experiment.

Mice inoculated with EDL933*Δstx:cat* or EDL933*cIind1* showed no signs of disease and were euthanized 3 weeks after inoculation. All of the mice inoculated with EDL933 became moribund prior to the scheduled necropsy date, and these mice were necropsied when they became moribund, between 10 and 18 days after inoculation (see Results). All animal experiments were conducted with the approval of the University of Michigan Animal Care and Use Committee.

At necropsy, cecal contents were cultured to determine bacterial colonization density. Quantitative counts were determined using LB agar plates containing appropriate antibiotics. Stx concentration in cecal contents was measured using a commercial kit (Premier) as previously described [7]. For histologic examination, right and left kidney were immersion-fixed in formalin, embedded in paraffin, cut in 5 micron sections, and stained with hematoxylin and eosin (Fig. 4). Kidney sections were scored by a single pathologist without knowledge of the source of the section. For quantitation, a midline section of the right renal cortex was examined in its entirety, and the number of 200× fields with tubular or glomerular lesions was recorded. Acute tubular necrosis was subjectively scored as mild, moderate, or severe.

For the SIVET experiment, animals were similarly infected with ∼10^6^ cfu of LB-cultured bacteria. Because of the deletion-substitutions in the 933W prophage, the SIVET strain does not express significant levels of Stx2. Details of the experiment procedure have been discussed above. In these experiments colony counts were obtained using LB plates containing either kanamycin (30 µg/ml) or cloramphenicol (9 µg/ml).

Statistics: Quantitative data were analyzed by Mann-Whitney U test. Multiple groups were compared by ANOVA and Fisher's Least Significant Difference.

### Construction of EDL933 SIVET strain

The design of SIVET [47] is based on Camilli and colleague's “Recombinase-based Reporter of Transcription (RIVET) system” [45], [46]. However, SIVET differs from RIVET in providing a selection for cells in which the assayed transcription occurred (Fig. 1-II). The first generation of EDL933 SIVET, was constructed similarly to the original K12 SIVET strain [47], [70] using recombineering [63], with a SpcR (this laboratory) derivative of pKD46 [65] supplying the λ Red functions. The 933W prophage was inactivated by elimination of genes controlling two critical components of phage growth, transcription and replication. The N gene, encoding a transcription regulator, was deleted and replaced with a KanR cassette. The *O* and *P* genes, encoding proteins involved in initiation of DNA replication [48], were replaced with the *tnpR* gene and *ampR* cassette. This was accomplished using a PCR product containing the *ampR* cassette and the sequence encoding the 168 variation of the γδ resolvase, *tnpR-168*, [46] with flanking sequences having homology to the 933W *cro* and *ren* genes (Fig. 1-I). These changes generated strain K11084 that, even though having a defective 933W prophage, is unable to survive treatment with an inducing concentration of mitomycin C. The cryptic prophage CP933V in EDL933, although defective, has nearly a complete lambdoid phage genome [41], leading us to suspect that its induction might be responsible for this sensitivity to mitomycin C. Therefore, we deleted the control region of CP933V rendering that prophage uninducible; the deletion included the putative repressor (*cI*) gene with immediate surrounding putative promoters, operators, genes, and relevant associated genetic material in a two-step process. A *cat-sacB* (CSB) cassette [71] with flanking ends having appropriate homologies to CP933V (primers 3 and 4, template K9685) was recombined into the targeted region, extending from *N* to *cII* (Fig. 1-I) in CP933V, generating strain K11114. The CSB inserted in CP933V was then replaced with a single-stranded DNA oligomer (oligo #1) [71], generating strain K11115. This strain survives the inducing levels of mitomycin C used in our studies.

Addition of the reporter cassette in a two-step procedure completed the construction of the EDL933 SIVET strain. First, K11161 was constructed by crossing a *cat* cassette (primers: 9 and 10, K10373 template) into the *lacZ* gene of K11115 providing homology for the next step. Second, K11173 was constructed by crossing the *cat::resC-tetR-resC::cat* cassette (primers7 and 8, K10449 template) into the inserted *cat* gene in K11161 with selection for tetracycline resistance.

This first EDL933 SIVET construct had to be modified because its constitutive expression of TetR made the bacteria sensitive to the in vivo environment. We therefore made the following changes using recombineering, λ Red functions were supplied by a hygromycin resistant derivative of pKD46 (pKD46hygR). The KanR cassette in the N gene was replaced by a *spcR* cassette and the selective *tetR* cassette in the *cat*::*resC*::*tet*::*resC*:*:cat* reporter was replaced by a *kanR* cassette yielding the *cat*::*resC*-*kan*-*resC*::*cat* reporter. To complete the process, the strain was cured of pKD46hygR yielding K11604, the SIVET strain used in the experiments reported here.

### Mitomycin C induction

The method used to obtain the results shown in figure 5 was essentially those outlined in Livny and Friedman [47]. Briefly, SIVET strain was grown ∼10^8^/ml in LB, made 2 µg/ml in mitomycin C, grown for 2 hrs, washed and resuspended in LB, grown for 4 hours, and dilutions of bacteria were plated on selective media.

### Induction Index

This metric provides a log_10_ scale readout that allows for a simplified comparison of results of different SIVET experiments. The calculations compare the ratio of CamR/KanR colonies at any given time relative to the starting ratio of CamR/KanR colonies. It is calculated as log_10_ [(CamR titer/KanR titer at any time after start of experiment)/(CamR titer/KanR titer at start of experiment)]. It follows that the Induction Index at the start would obviously be 0; i.e., log_10_ 1 (starting ratio/starting ratio).

### Effect of induction of *cIind1* mutant on lysis

Overnight cultures of O157:H7 and the *cIind1* derivative grown in LB broth were diluted 1∶100 in LB and grown to early log phase. Each were divided into two aliquots, one untreated and the other treated with 2 µg/ml of mitomycin C. Samples, 200 µl, were placed in a 96 well plate and grown at 37° with OD_600_ read at 30 minute intervals in the SpectraMax 250 (Micro Devices).

## Supporting Information

Figure S1 **Mathematical modeling of Induction Index.** To determine what would be expected if the contribution to the CamR population by spontaneous induction during growth of the SIVET population were unimpeded, we used mathematical modeling to predict what such an unimpeded expansion would look like. The change in the ratio of CamR/KanR, due to spontaneous induction during bacterial growth is calculated and displayed as an Induction Index over a range of population doublings reaching 1000. This allows comparison with the results obtained by experimentation. The CamR bacteria resulting from the conversion of KanR bacteria are added to the growing CamR culture which is determined by the standard exponential growth equation taking into consideration, as we have shown, that the two populations grow at the same rate. The model is further expanded to take into account possible effects of phenotypic lag, the period following induction needed for the newly converted CamR bacteria to recover from the damage inflicted during the resulting SOS response. The top line shows the expected increase in the ratio CamR/KanR populations due to spontaneous induction, assuming the conversion was free of any factors impeding the conversion number (see text for detailed discussion of possible factors). The lines below show a range of possible delays due to phenotypic lag prior to the first doubling. The starting ratio is set to 1, or an Induction Index of 0 on the log scale. Other factors possibly reducing the actual conversion number were not considered in the modeling.
(TIF)

Text S1 **Supplementary methods.** Experimental procedures describing the mathematical modeling of Induction Index.
(DOCX)
