# Supplementary figures and images for: Prophage Induction Is Enhanced and Required for Renal Disease and Lethality in an EHEC Mouse Model
Source: PLoS Pathog. 2013 Mar 28;9(3):e1003236. doi: 10.1371/journal.ppat.1003236 (PMC3610611; doi:10.1371/journal.ppat.1003236)

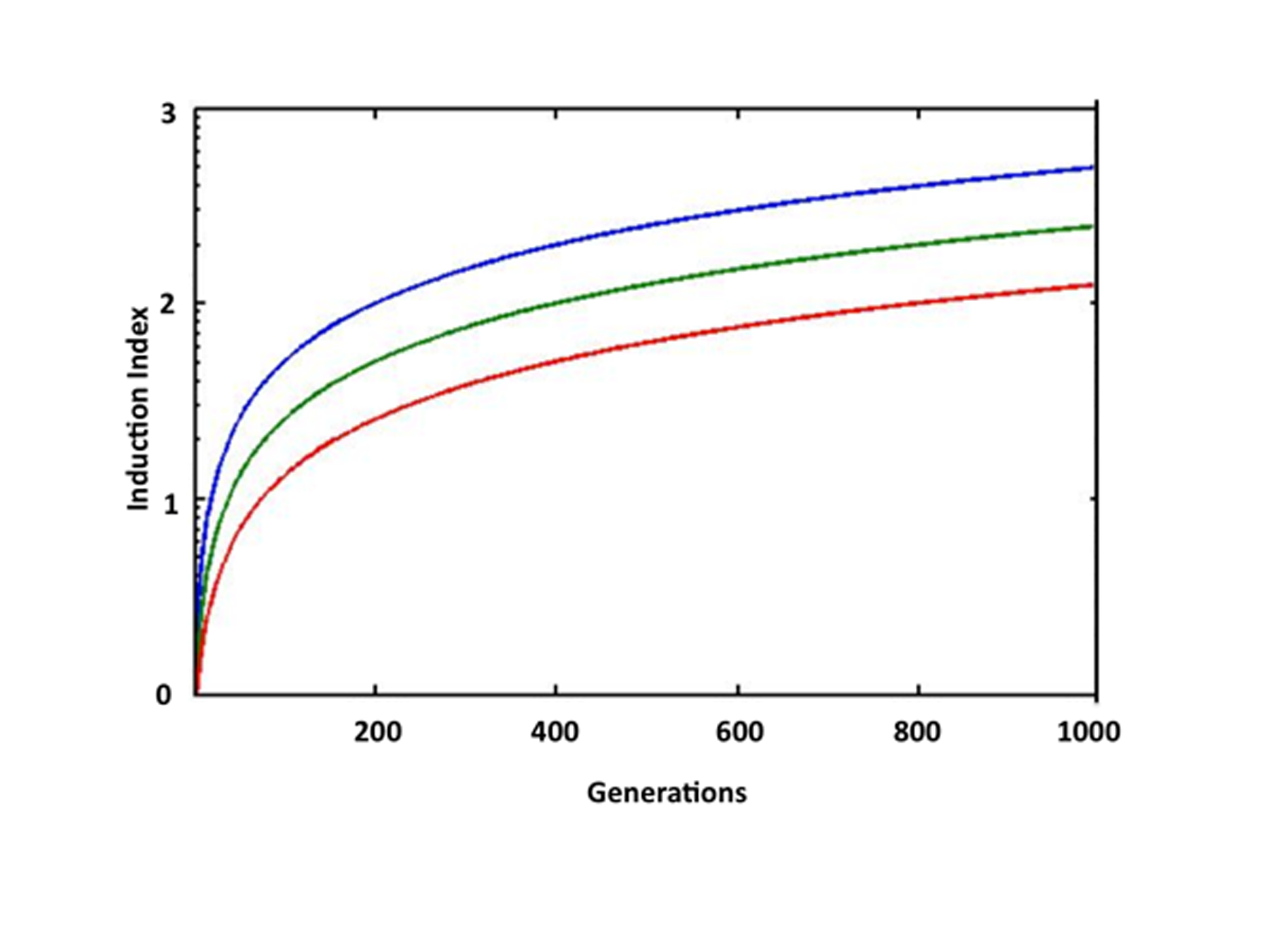

Supplement: Figure S1 — Mathematical modeling of Induction Index. To determine what would be expected if the contribution to the CamR population by spontaneous induction during growth of the SIVET population were unimpeded, we used mathematical modeling to predict what such an unimpeded expansion would look like. The change in the ratio of CamR/KanR, due to spontaneous induction during bacterial growth is calculated and displayed as an Induction Index over a range of population doublings reaching 1000. This allows comparison with the results obtained by experimentation. The CamR bacteria resulting from the conversion of KanR bacteria are added to the growing CamR culture which is determined by the standard exponential growth equation taking into consideration, as we have shown, that the two populations grow at the same rate. The model is further expanded to take into account possible effects of phenotypic lag, the period following induction needed for the newly converted CamR bacteria to recover from the damage inflicted during the resulting SOS response. The top line shows the expected increase in the ratio CamR/KanR populations due to spontaneous induction, assuming the conversion was free of any factors impeding the conversion number (see text for detailed discussion of possible factors). The lines below show a range of possible delays due to phenotypic lag prior to the first doubling. The starting ratio is set to 1, or an Induction Index of 0 on the log scale. Other factors possibly reducing the actual conversion number were not considered in the modeling. (TIF) [file ppat.1003236.s001.tif]
