# Supplementary material for: Prophage Induction Is Enhanced and Required for Renal Disease and Lethality in an EHEC Mouse Model
Source: PLoS Pathog. 2013 Mar 28;9(3):e1003236. doi: 10.1371/journal.ppat.1003236 (PMC3610611; doi:10.1371/journal.ppat.1003236)
Supplement: Text S1 — Supplementary methods. Experimental procedures describing the mathematical modeling of Induction Index. (DOCX) [file ppat.1003236.s002.docx]

**Supporting information**

**Experimental Procedures**

Modeling calculations:

NOTE: for this discussion A= KanR colonies and B = CamR colonies

To model the expected growth behavior of the two colonies A and B, we created a standard discrete geometric model of bacterial growth of A and a combined discrete geometric and induced growth model for B. So that at any time or generation k, the population of colonies in A which we denote
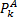

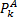
 is:


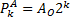


where
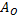

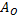
 is the initial population of colony A. And the population of colonies in B which we denote
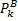

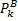
 is:


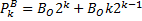


where
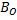

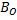
 is the initial population of colony B,
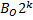

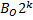
 is the geometric growth of the B colony from the initial population and
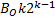

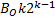
 is the induced population from A. (The generation, *k*, is divided by 2 to avoid counting twice the addition to the CamR population when the newly formed CamR bacterium resulting from prophage induction divides. This because following division only one of the two daughter bacterium would add to the CamR colony count in the new generation since one of the daughter bacterium would already have been accounted for in the previous generation.)

Then we look at the ratio of the population of B to that of A and normalize by the initial value of the ratio, we have


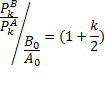


We can also account for the possible delay in the induced growth of B where d is the number of

time steps that are required for B to be repaired and begin growing


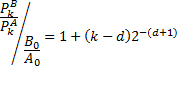


We can program this equation in MATLAB from MathWorks as

for m=1 : 3

                d=m-1;

      for k=1 : n;

            if   (k-d) >=0

              ratio (k,m)=1 + (k-d) *2 ^(-d-1);

else

ratio (k,m) =1;

end

end

end
